# Supplementary material for: Loss of NEDD8 in cancer cells causes vulnerability to immune checkpoint blockade in triple-negative breast cancer
Source: Nat Commun. 2024 Apr 27;15:3581. doi: 10.1038/s41467-024-47987-x (PMC11055868; doi:10.1038/s41467-024-47987-x)
Supplement: Supplementary file 1 — Supplementary Information [file 41467_2024_47987_MOESM1_ESM.pdf]

Supplemental figures and figure legends

Supplementary Figure 1.

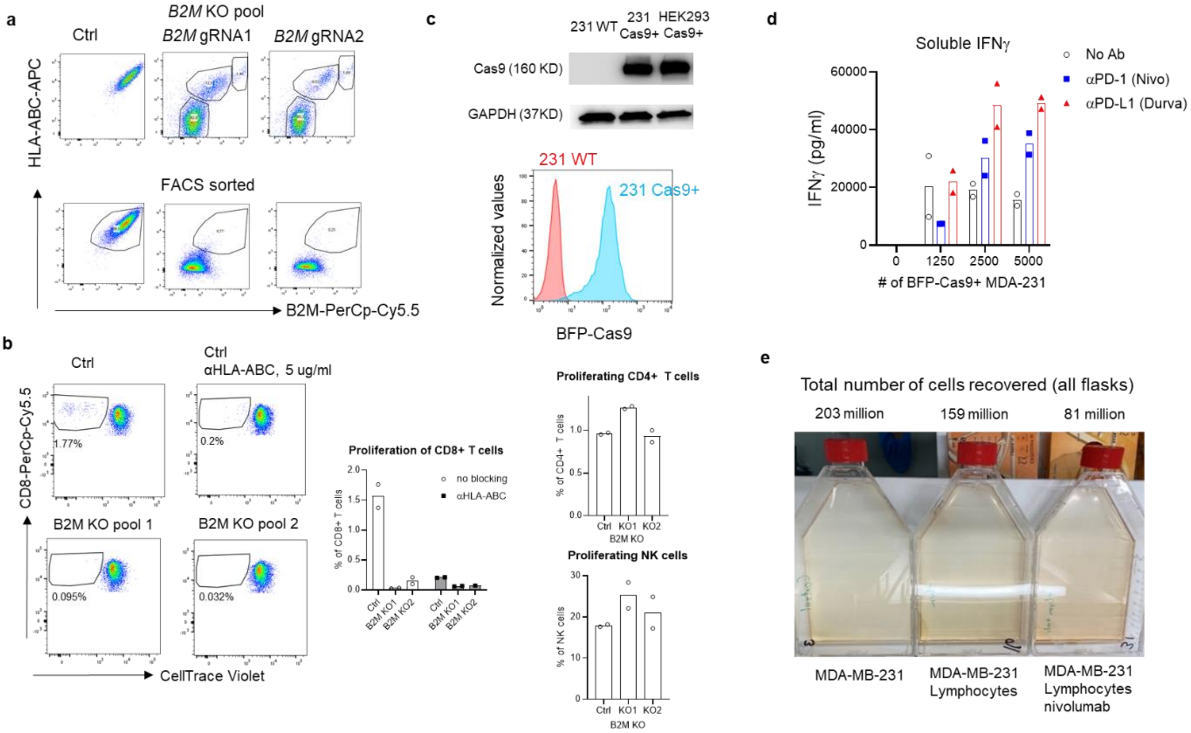

Supplementary Figure 1. a Human *B2M* gene was deleted in MDA-MB-231 by transfecting ribonucleoprotein (RNP) complexes using electroporation. HLA-ABC/B2M negative cells were enriched using FACS sorting. Representative plot of 3 independent repeats was shown. **b** CTV-pulsed primary human lymphocytes were co-cultured with control (ctrl) or *B2M* knockout (KO) MDA-MB-231 cells +/- a blocking antibody against HLA-ABC. The resulted immune cell activation was quantified using flow cytometry on day 5. Representative data of 3 independent repeats. **c** A vector encoding the Cas9 protein was transduced in MDA-MB-231 wild-type (WT) cells and enriched using FACS sorting. One experiment was performed. **d** Immune activation primed by the Cas9-expressing MDA-MB-231 cells was tested in tumor-immune co-culture system (TICS). Representative data from 2 independent repeats. **e** Pictures of flasks and final cancer cell numbers at the end of the CRISPR screen to demonstrate reduced medium consumption due to lymphocyte activation. One representative flask of 15 flasks in each group. Source data are provided as a source data file.

Supplementary Figure 2.

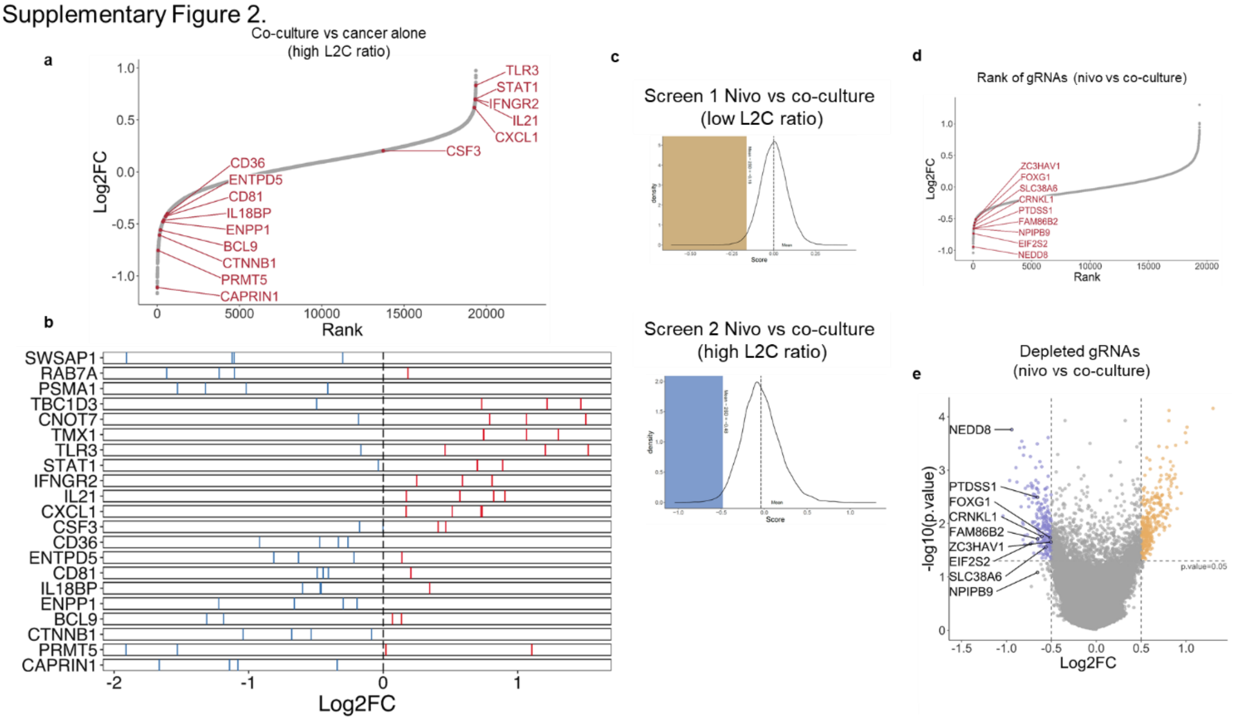

Supplementary Figure 2. a Demonstration of enriched (right) or depleted (left) genes and **b** gRNA performance when comparing co-culture or MDA-MB-231 cells cultured alone; depleted gRNAs (blue), enriched gRNAs (red). **c** Cut-off used to select depleted genes from both CRISPR screens in tumor-immune co-culture system (TICS). The 9 commonly depleted genes in CRISPR screens using TICS, shown in **d** rank order or **e** in a volcano plot; blue: depleted gRNAs, orange: enriched gRNAs.

Supplementary Figure 3.

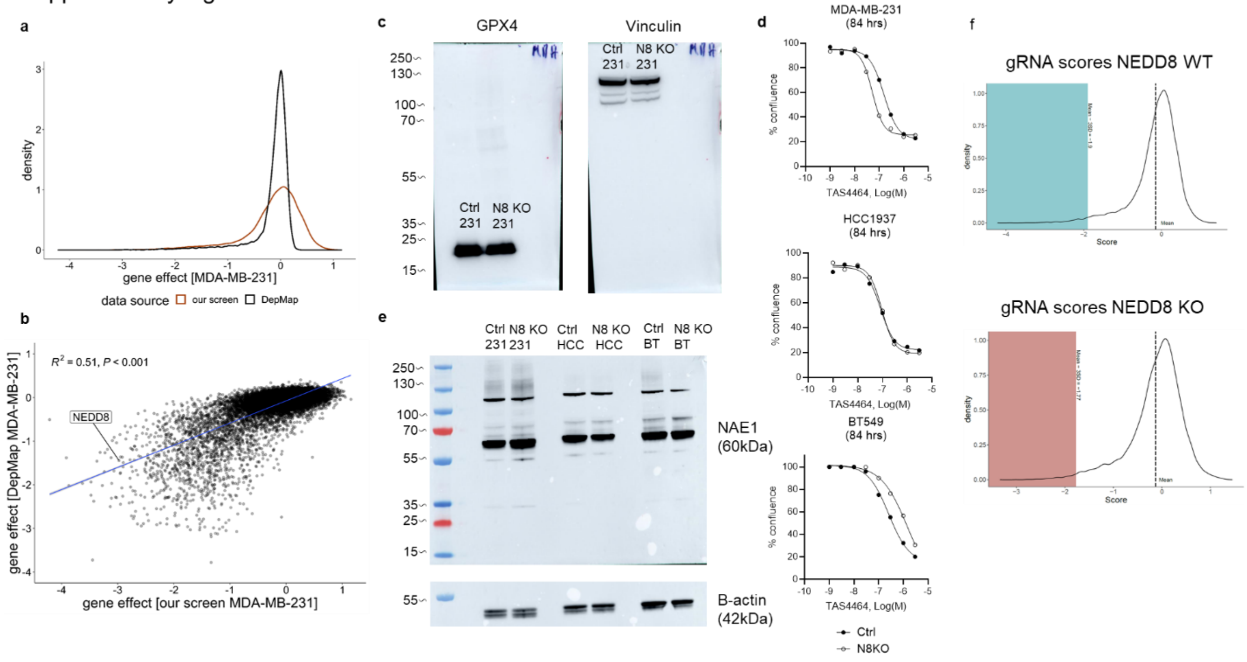

Supplementary Figure 3. a Distribution and **b** correlation of gene effects in genome-wide CRISPR screens on wild-type (WT) MDA-MB-231 cells in our laboratory and a previous screen from DepMap. **c** Expression of GPX4 was measured in MDA-MB-231 control (ctrl) and *NEDD8* knockout (KO) cells using Western Blotting. Representative image of 3 independent repeats was shown. **d** Ctrl or *NEDD8* KO lines from 3 human triple-negative breast cancer (TNBC) cell lines, i.e. MDA-MB-231, HCC1937 and BT549, were treated with TAS4464 at increasing concentrations. Cell proliferation was monitored using a live-cell imaging system and dose response curves were calculated at 84 hours. Representative experiment of 3 independent repeats was shown. **e** Expression of NAE1 in Ctrl /KO cell line pairs generated from 3 human breast cancer cell lines as above. Representative image from 2 independent repeats were shown. **f** Cut-off used to select depleted genes from CRISPR screens using WT or *NEDD8* KO MDA-MB-231 cells. Source data are provided as a source data file.

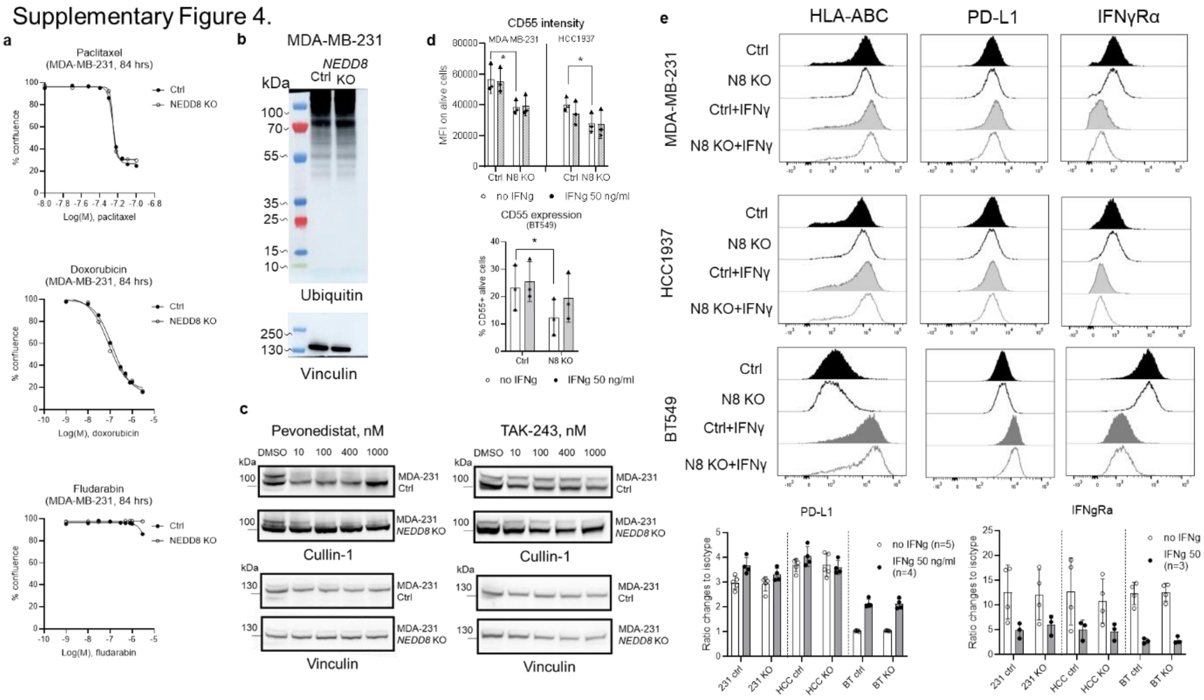

Supplementary Figure 4. a Control (ctrl) or *NEDD8* knockout (KO) MDA-MB-231 cells were treated with chemotherapeutic drugs paclitaxel, doxorubicin or fludarabin at increasing concentrations. Cell proliferation was monitored using a live-cell imaging system and dose response curves were calculated at 84 hours. Representative experiment of 2 independent repeats was shown. **b** Total ubiquitination was tested in control or *NEDD8* KO MDA-MB-231 cells using Western Blotting. Representative image of 2 independent repeats was shown. **c** Control or *NEDD8* KO cells were treated with 1000, 400, 100, 10 nM pevonedistat or TAK-243 for 24 hours. Expression of cullin-1 was determined using Western Blotting. DMSO (0.1%) was used as controls. Representative image of 2 independent repeats. Ctrl or KO lines from 3 human triple-negative breast cancer (TNBC) cell lines were cultured with or without 50 ng/ml interferon γ (IFNγ). Cells were harvested after 24 hours and surface expression of **d** CD55 (n=3) or **e** PD-L1 (no IFNg, n=5; +IFNg, n=4) and IFNγRα (no IFNg, n=4; +IFNg, n=3) were quantified using flow cytometry (Mean±SD). Each dot represented an independent experiment. Representative histograms of HLA-ABC, PD-L1 or IFNgRa were shown. Source data are provided as a source data file.

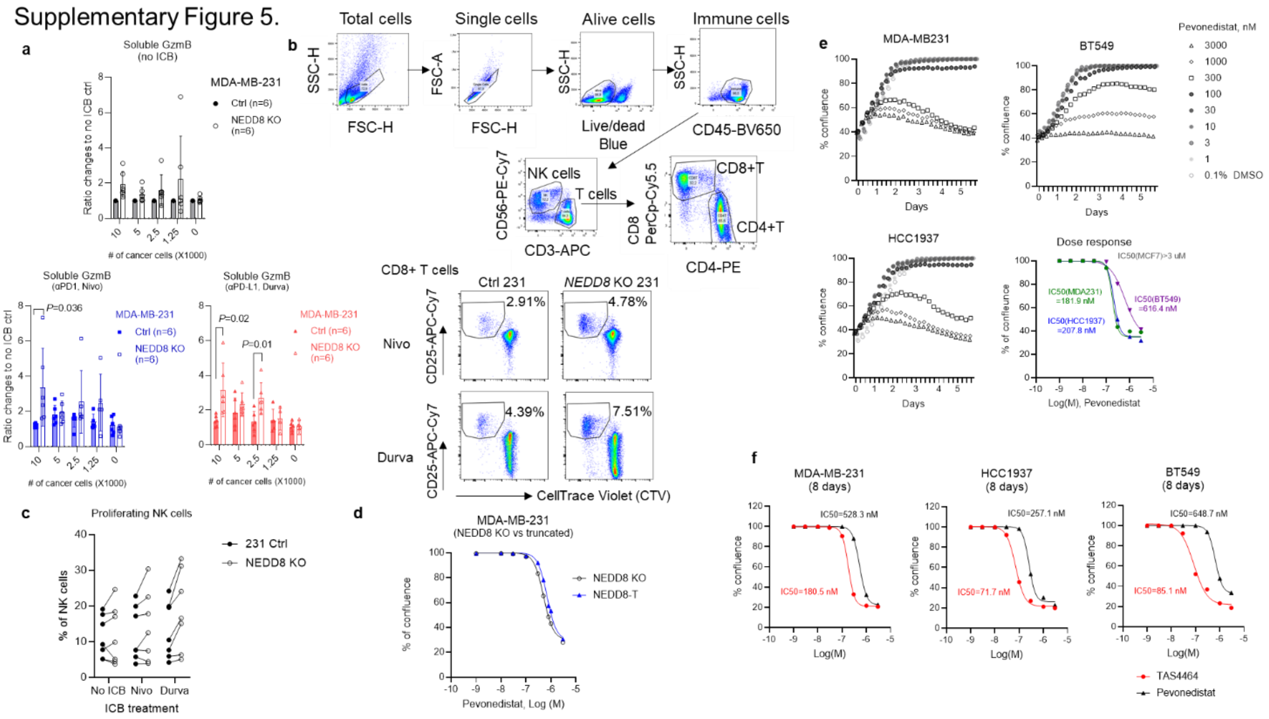

Supplementary Figure 5. a Control (ctrl) or NEDD8 knockout (KO) MDA-MB-231 cells were co-cultured with primary human lymphocytes +/- 10 μg/ml nivolumab or durvalumab. Release of granzyme B (GzmB) to the culture supernatants was quantified using ELISA on day 5 from 6 independent donors. Mean±SD and unpaired 2-tailed T-test. **b** FACS gating strategy and representative plots of at least 3 independent repeats to show proliferation of CD8+ T cells co-cultured with control or KO MDA-MB-231 cells +/- 10 μg/ml nivolumab or durvalumab on day 5. **c** CellTrace Violet (CTV)-pulsed primary human lymphocytes were co-cultured with ctrl or *NEDD8* KO MDA-MB-231 cells +/- 10 μg/ml nivolumab or durvalumab. Proliferation of NK cells was determined using flow cytometry on day 5 from 6 independent donors. The lines connected values from the same lymphocyte donor. **d** NEDD8 KO MDA-MB-231 cells or KO cells transduced with the truncated NEDD8 protein (NEDD8-T), were treated with pevonedistat at increasing concentrations. Cell proliferation was monitored using a live-cell imaging system and dose response curves were calculated at 84 hours. Representative experiment of 2 independent repeats was shown. **e** Three human triple-negative breast cancer (TNBC) cell lines, i.e. MDA-MB-231, HCC1937 or BT-549, were treated with increasing concentrations of

pevonedistat and cell proliferation was measured using a live-cell imaging system. Dose-response curves were generated at 84 hours. Representative experiment of 3 independent repeats. **f** Inhibitory effects of pevonedistat or TAS4464 was compared on the three human TNBC cell lines using a live-imaging system. Representative experiment from 3 independent repeats was shown. Source data are provided as a source data file.

Supplementary Figure 6.

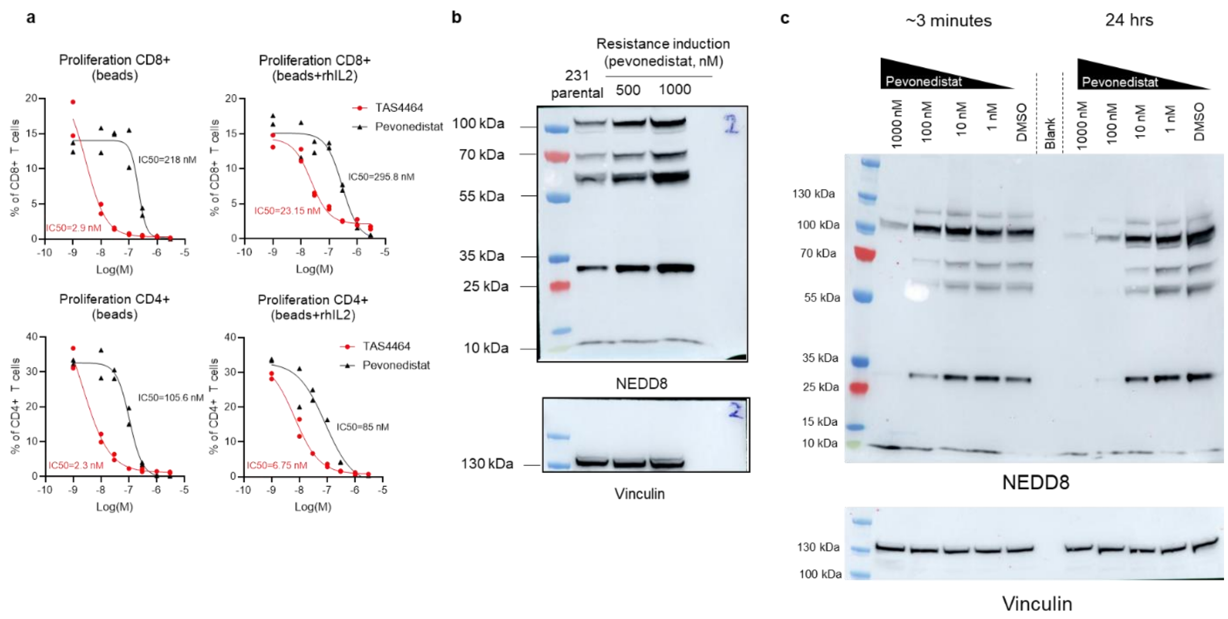

Supplementary Figure 6. a CellTrace Violet (CTV)-labelled primary human lymphocytes were cultured with microbeads coated with CD3/28 antibodies +/- rhIL2, in presence of increasing concentrations of pevonedistat or TAS4464. Cells were harvested after 5 days and proliferation of CD8+ or CD4+ T cells was analyzed by flow cytometry. Representative experiment of 3 independent donors. **b** Pevonedistat-resistant MDA-MB-231 cells were generated by chronic exposure of parental cells to either 500 nM or 1000 nM of pevonedistat. Expression of NEDD8 or neddylated proteins were measured in parental or resistant cells using Western Blotting. Representative image of 2 independent repeats was shown. **c** Pevonedistat-resistant MDA-MB-231 cells were treated with increasing concentrations of pevonedistat. Cells were harvested immediately after compound addition or at 24 hours. Expression of NEDD8 or neddylated proteins was measured using Western Blotting. Representative image of 2 independent repeats was shown. Source data are provided as a source data file.

Supplementary Figure 7.

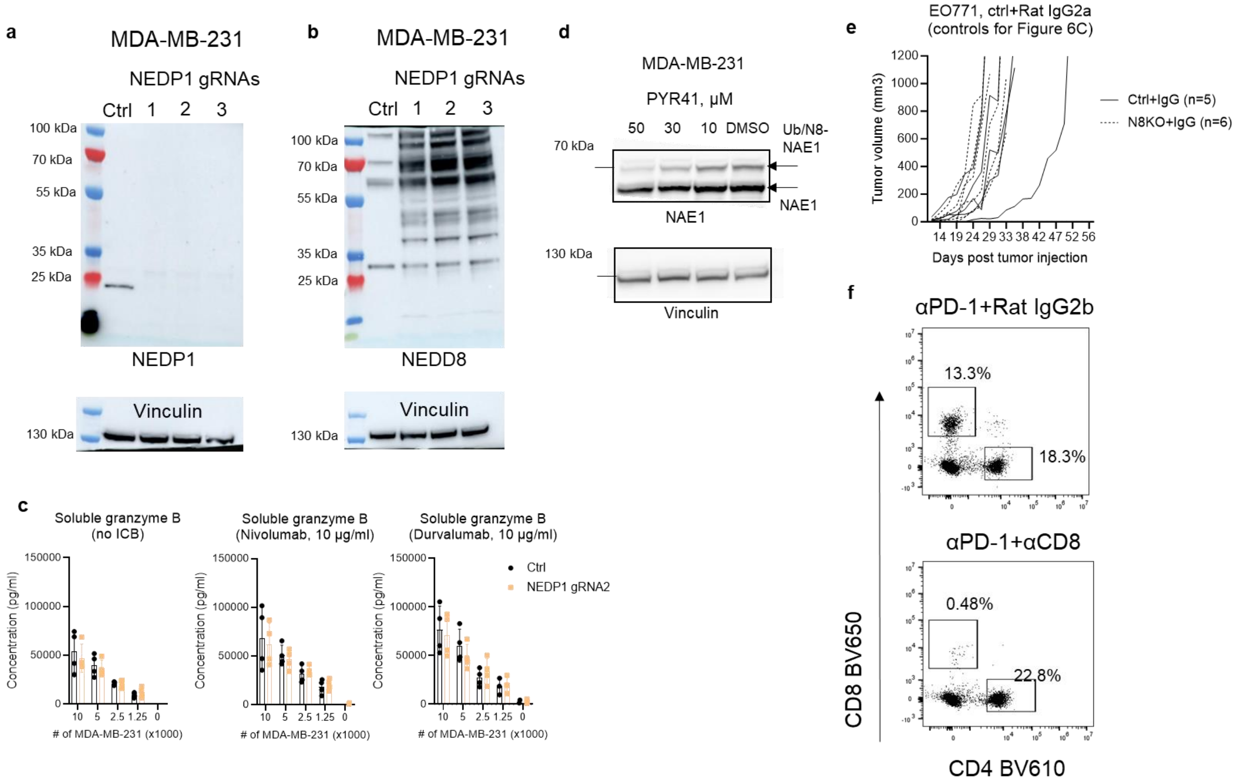

Supplementary Figure 7. a *NEDP1* gene was deleted by CRISPR/Cas9 in MDA-MB-231 cells. Control (ctrl) cells were created at the same time by transfecting ribonucleoprotein (RNP) complexes without the gene-targeting crRNA. **b** Protein neddylation was measured in control and *NEDP1* knockout (KO) cells were measured using Western Blotting. Representative image of 3 independent repeats. **c** Control or *NEDP1* KO MDA-MB-231 cells were co-cultured with primary human lymphocytes in tumor-immune co-culture system (TICS) +/- 10 µg/ml nivolumab. Soluble granzyme B levels were quantified using ELISA on day 5 with 4 independent donors. **d** Control or *NEDD8* KO MDA-MB-231 cells were treated with 50, 40, 30, 20 or 10 µM of the UBA1 inhibitor, PYR41, in 1% DMSO for 24 hours. Expression of NAE1 was measured using western blotting. Representative image of 2 independent repeats. **e** Control or *Nedd8* KO EO771 cells were injected subcutaneously in female C57BL/6NTac mice and treated with a Rat IgG2a isotype control antibody. Tumor volumes were recorded and compared. **f** Wild-type (WT) or *Nedd8* KO EO771 cells were injected subcutaneously in female C57BL/6NTac mice. CD8+ T cells were depleted using an antibody and frequencies of CD8+

T cells in the spleens were measured using flow cytometry. Representative plot of at least 3 independent mice. Source data are provided as a source data file.

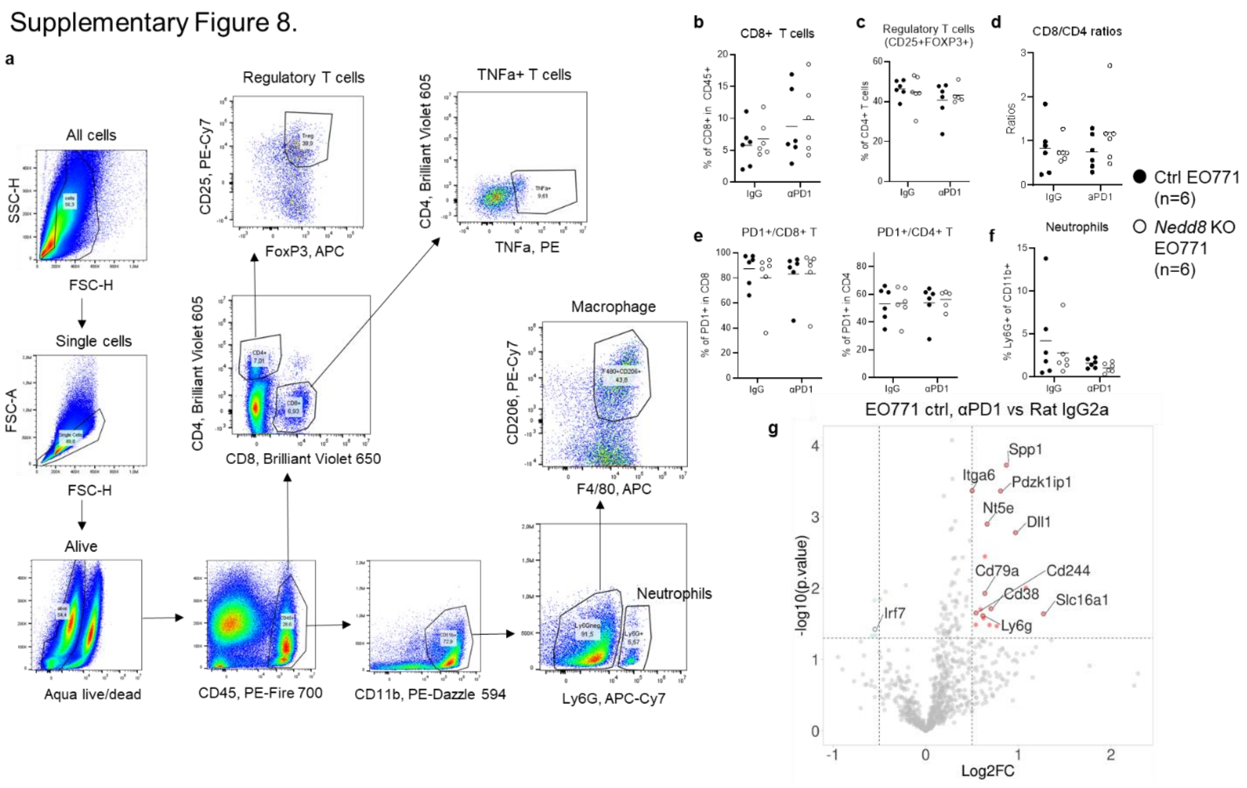

Supplementary Figure 8. a Gating strategy for T cells and myeloid cells using single cells isolated from EO771 tumor-bearing mice. Frequencies of **b** CD8+ T cells, **c** regulatory T cells (CD25+FoxP3+CD4+ T cells), **d** ratios between CD8+ and CD4+ T cells, **e** PD1+ T cells or **f** Ly6G+ neutrophils were compared among groups. Each dot represented cells from an individual mouse of 6 mice per group. **g** Differentially expressed mRNAs were compared between EO771 tumors treated with the PD-1 blocking antibody or the isotype control. Cut-off: Log2FC>0.5 and *P*<0.05, unpaired 2-taíled T test. Up-regulated (red) and down-regulated (blue) mRNAs were shown in a volcano plot. Source data are provided as a source data file.

Supplementary Tables

Supplementary Table 1: Antibodies

Name	Clone	Application	Product information
Anti-human/mouse NEDD8	Rabbit monoclonal [Y297] IgG	WB	Abcam/ab81264<br>1:2000
Anti-human HSPC150/UBE2T	Rabbit monoclonal [EPR9446] IgG	WB	Abcam/ab140611<br>1:5000
Anti-human/mouse NAE1/APPBP1	Rabbit IgG, AG6672	WB	Proteintech/14863-1-AP<br>1:1500
Anti-human GPX4	Rabbit monoclonal [EPNCIR144] IgG	WB	Abcam/ab125066<br>1:1000
Anti-human NEDP1	Polyclonal antibody	WB	Thermo Fisher Scientific /PA5-31033<br>1:1000
Anti-human Ubiquitin	Monoclonal antibody [GT7811]	WB	Thermo Fisher Scientific /MA5-42345<br>1:1200
Anti-human Cullin 1	Monoclonal antibody [2H4C9]	WB	Thermo Fisher Scientific /32-2400<br>1 µg/ml
Anti-human CDT1	Rabbit polyclonal antibody	WB	Proteintech 29766-1-AP-20UL<br>1:2500
Anti-Rabbit IgG HRP-linked Antibody	Goat IgG	WB	Cell Signaling Technology/7074S<br>1:6000
Anti-mouse IgG HRP-linked-Antibody	Horse IgG	WB	Cell Signaling Technology/7076S<br>1:6000
Anti-human/mouse Vinculin	nVin-1, mouse IgG1	WB	Sigma Aldrich/V9131<br>1:2000
Anti-human/mouse β-Actin (ACTBD11B7)	Mouse monoclonal IgG	WB	Santa Cruz Biotechnology/sc-81178<br>1:2500
Anti-CRISPR-Cas9 antibody [7A9-3A3]	Mouse monoclonal IgG1	WB	ABCAM/ab191468<br>3 µg/ml
Anti-GAPDH−Peroxidase antibody	GAPDH-71.1, mouse IgM	WB	Sigma Aldrich/G9295<br>1 µg/ml
OPDIVO (nivolumab) anti-PD-1	Human monoclonal IgG4	In vitro block	Bristol-Myers Squibb
IMFINZI (durvalumab) anti-PD-L1	Human monoclonal IgG1, k	In vitro block	AstraZeneca
Anti-human HLA-ABC	W6/32, mouse IgG2a, k	In vitro block	Biolegend/311428
FITC anti-human CD38	HIT2, mouse IgG1, k	FACS	Biolegend/303504
Brilliant Violet 650 anti-human CD45	HI30, mouse IgG1, k	FACS	Biolegend/304044
APC anti-human CD55	JS11, mouse IgG1, k	FACS	Biolegend/311311
APC anti-human HLA-ABC	W6/32, mouse IgG2a, k	FACS	Biolegend/311410
PE anti-human CD119 (IFN-γ R a chain)	GIR-208, mouse IgG1, k	FACS	Biolegend/308606
FITC anti-human CD274 (B7-H1,PD-L1)	MIH2, mouse IgG1, k	FACS	Biolegend/393606
APC/Cyanine 7 anti-human HLA-DR	L-243, mouse IgG2a, k	FACS	Biolegend/307618
PerCp-Cy5.5 anti-human B2M	2M2, mouse IgG1, k	FACS	Biolegend/316315
APC anti-human CD3	HIT3a, mouse IgG2a, k	FACS	Biolegend/300312

PE anti-human CD4	OKT4, mouse IgG2b, k	FACS	Biolegend/317410
PerCp-Cy5.5 anti-human CD8	RPA-T8, mouse IgG1, k	FACS	Biolegend/301032
PE-Cy7 anti-human CD56	HCD56, mouse IgG1, k	FACS	Biolegend/318318
APC-Cy7 anti-human CD25	BC96, mouse IgG1, k	FACS	Biolegend/302614
PE anti-mouse CD4	RM4, rat IgG2a, k	FACS	Biolegend/100512
PE-Cy7 anti-mouse CD25	PC61, rat IgG1, λ	FACS	Biolegend/102016
PE anti-mouse TNF alpha	MP6-XT22, rat IgG1, k	FACS	Thermo Fisher Scientific/12-7321-81
PerCP-Cy5.5 anti-mouse CD3	17A2, rat IgG2b, k	FACS	Biolegend/100218
Brilliant Violet 650 anti-mouse CD8a	53-6.7, rat IgG2a, k	FACS	Biolegend/100742
PE-Fire 700 anti-mouse CD45	30-F11, rat IgG2b, k	FACS	Biolegend/103178
APC-Cy7 anti-mouse Ly6G	1A8, rat IgG2a, k		Biolegend/127624
PE-Dazzle 594 anti-mouse/human CD11b	M1/70, rat IgG2b, k	FACS	Biolegend/101256
APC anti-mouse F4/80	BM8, rat IgG2a, k	FACS	Biolegend/123116
PE-Cy7 anti-mouse CD206	C068C2, rat IgG2a k	FACS	Biolegend/141720
Anti-Mo CD16/CD32	93, rat IgG2a, λ	FACS , FcR blocker	Thermo Fisher Scientific/14-0161-85
Anti-mouse PD-1 (CD279)	RMP1-14, rat IgG2a, k	In vivo block	BioXcell/BE0146
Rat IgG2a isotype9	2A3, rat IgG2a k	In vivo block	BioXcell/BE0089
Anti-mouse CD8a	2.43, rat IgG2b**, k**	In vivo block	BioXcell/BE0061
Rat IgG2b isotype	LTF-2, rat IgG2b, k	In vivo block	BioXcell/BE0090

Supplementary Table 2: other reagents

Name	Application	Product information
QIAmp DNA Blood Maxi kit	DNA isolation	QIAGEN/51194
Lymphoprep	Cell isolation	StemCell/07851-07861
SepMate tubes	Cell isolation	StemCell/85450
CD14+ positive selection kit	Cell isolation	StemCell/17858
Red blood cell lysis buffer	Cell isolation	Biolegend/420301
RNeasy Mini kit	mRNA isolation	Qiagen/74104
Neon transfection system kit	CRISPR KO	Invitrogen/MPK10025
Alt-R® S.p. Cas9 Nuclease V3	CRISPR KO	IDT/1081058
TracrRNA	CRISPR KO	IDT/1072534
IDTE buffer	CRISPR KO	IDT/11-01-02-02
Nuclease free duplex buffer	CRISPR KO	IDT/11-01-03-01
IFNg ELISA Kit	ELISA	Biolegend/430104
ELISA Flex: Human IFN-γ (HRP)	ELISA	MabTech/3220-1H-20
Granzyme B ELISA Kit	ELISA	MabTech/3485-1H-20
Blocker BSA (10%) in PBS	ELISA	Thermo Scientific/37525
1-Step ultra TMB-ELISA	ELISA	Thermo Scientific/34028
ELISA stop solution	ELISA	Thermo Fisher Scientific/SS04
Tween 20	ELISA/WB	Fisher bioreagents/9005-64-5
FoxP3/transcription factor staining buffer set	FACS	eBioscience/00-5523-00
Aqua fixable live/dead marker	FACS	Invitrogen by Thermo Fisher Scientific/L34966A
CellTrace violet	FACS	Invitrogen by Thermo Fisher Scientific/C34557
Tumor dissociation kit, mouse	Tissue digest	Miltenyi Biotech/130-096-730
GentleMacs C-tubes	Tissue digest	Miltenyi Biotech/130-093-237
MACS smartstrainers	Tissue digest	Miltenyi Biotech/130-110-916
Mycoplasma detection kit	Cell culture	Lonza/LT07-218
IMDM medium	Cell culture	Thermo Fisher Scientific /12440-053
Heat inactivated FBS	Cell culture	Thermo Fisher Scientific /10500-064
PenStrep	Cell culture	Thermo Fisher Scientific /15140-122
Versene	Cell culture	Thermo Fisher Scientific /15040-033
PBS	Cell culture	Thermo Fisher Scientific /20012-027
Trypan Blue Solution, 0.4%	Cell counting	VWR/K940
WB gels (NuPAGE 4-12% Bis-Tris Gel)	WB	Invitrogen by Thermo Fisher Scientific/NP0321BOX
Protein Ladder	WB	Thermo Scientific/26619
iBlot 2NC Regular Stacks	WB	Invitrogen by Thermo Fisher Scientific/IB23001
MOPS SDS Running Buffer (20x)	WB	Novex by life technologies/NP0001
Transfer Buffer (20x)	WB	Novex by life technologies/NP0006-1
LDL Sample buffer (4x)	WB	Novex by life technologies/B0007
SKIM Milk POWDER	WB	OXOID/LP0033
Ponceaus S solution for electrophoresis (0.2%)	WB	Serva/33427.01
Pico substrate	WB	Thermo Scientific/34580
Femto substrate	WB	Thermo Scientific/34095
RIPA Buffer	WB	Thermo Scientific/89900
BCA Protein Assay Kit	WB	Thermo Scientific/23225
Protease and Phosphatase Inhibitor Cocktail	WB	Thermo Scientific/78446
Dimethyl Sulfoxide (DMSO)	In vitro	Fisher bioreagents/67-68-5
ImmunoCult Human CD3/CD28 T Cell Activator	In vitro stim	Stemcell/10971
rhIFNγ	In vitro	Peprotech/AF-300-02
rhIL2	In vitro	Peprotech/AF-200-02
Pevonedistat (MLN4924)	Inhibitor	MedChemExpress/HY-70062/CS-0348

TAS4464 (hydrochloride)	Inhibitor	MedChemExpress/HY-128586A/CS-0095953
TAK-243 (MLN7243)	Inhibitor	Selleck Chemicals/S8341-2MG
Fludarabine	Inhibitor	MedChemExpress/21679-14-1
Doxorubicin (Andriamycin) HCI	Inhibitor	Selleckchem/S1208
PYR-41	Inhibitor	MedChemExpress/418805-02-41
Paclitaxel	Inhibitor	Cell signaling/9807S
pHAGE-EF1-dCas9-KRAB	Plasmid	Addgene #50919
psPAX2	Plasmid	Addgene #12260
pCMV-VSVG	Plasmid	Addgene #8454
FuGENE 6	Transfection	Promega/E2691
BsrGI	Buffer	New England BioLabs/R0575S

Supplementary Table 3: sequences

Name	Sequence
Hu NEDD8 crRNA4	*TGACATTGAACCTACAGACA*
Hu B2M crRNA1	*CGTGAGTAAACCTGAATCTT*
Hu B2M crRNA2	*AAGTCAACTTCAATGTCGGA*
Mo NEDD8 crRNA5	*TCTAAAGGTGGAGCGAATCA*
Mo NEDD8 crRNA8	*AGACATCGAACCCACAGACA*
Carrier DNA	*CCAGCAGAACACCCCCATCGGCGACGGCCCCGTGCTGCTGCCCGACAACCACTACCTGAGCACCCAGTCCGCCCTGAGCAAAGACCCCAACGAGA*
NEDD8trunc gBlock in bold	*GTGTGGTGGAATTCTGCAGATATCAACAAGTTTGTACgccaccATGCTAATTAAAGTGAAGACGCTGACCGGAAAGGAGATTGAGATTGACATTGAACCTACAGACAAGGTGGAGCGAATCAAGGAGCGTGTGGAGGAGAAAGAGGGAATCCCCCCACAACAGCAGAGGCTCATCTACAGTGGCAAGCAGATGAATGATGAGAAGACAGCAGCTGATTACAAGATTTTAGGTGGTTCAGTCCTTCACCTGGTGTTGGCTCTGAGATGAGTACAAAGTGGTTGATATCCAGCACAGTGGCG*
Hu NEDP1 crRNA1	*CCCCGTAGTCTTGAGTTACA*
Hu NEDP1 crRNA2	*GTTACATGGACAGTCTACTG*
Hu NEDP1 crRNA3	*CATCAATGATAACTCCAACC*
Hu NAE1/APPBP1 crRNA3	*TCAAAGAAGCAGTATCGGCA*

Supplementary Table 4: Detected peptides from the proteomics assay

**Sample**	**No. Identified proteins**
Ctrl-1	2554
Ctrl-2	2520
Ctrl-3	2305
Ctrl-4	2378
NEDD8 KO-5	2295
NEDD8 KO-6	2363
NEDD8 KO-7	2383
NEDD8 KO-8	2364
