# Supplementary material for: Loss of NEDD8 in cancer cells causes vulnerability to immune checkpoint blockade in triple-negative breast cancer
Source: Nat Commun. 2024 Apr 27;15:3581. doi: 10.1038/s41467-024-47987-x (PMC11055868; doi:10.1038/s41467-024-47987-x)
Supplement: Supplementary file 3 — Description of Additional Supplementary Files [file 41467_2024_47987_MOESM3_ESM.pdf]

Description of Additional Supplementary Files

Supplementary Data 1. A summary of gRNA counts from genome-wide CRISPR screens. Two screens were done with MDA-MB-231 control cells co-cultured with primary human lymphocytes+/-nivolumab. One screen was done with either control or NEDD8 KO MDA-MB-231 cells.

Supplementary Data 2. This file contains the processed results of label-free proteomics analysis with either MDA-MB-231 control or NEDD8 KO cells. Each cell line contains 4 replicates.

Supplementary Data 3. The results are from the nanostring analysis of single cells isolated from control or *Nedd8* KO EO771 tumors treated with either an anti-PD1 antibody or the isotype control.
