# Supplementary material for: Loss of NEDD8 in cancer cells causes vulnerability to immune checkpoint blockade in triple-negative breast cancer
Source: Nat Commun. 2024 Apr 27;15:3581. doi: 10.1038/s41467-024-47987-x (PMC11055868; doi:10.1038/s41467-024-47987-x)
Supplement: Supplementary file 7 — Reporting Summary [file 41467_2024_47987_MOESM7_ESM.pdf]

nature portfolio

Corresponding author(s): Yumeng Mao

Last updated by author(s): Apr 5, 2024

Reporting Summary

Nature Portfolio wishes to improve the reproducibility of the work that we publish. This form provides structure for consistency and transparency in reporting. For further information on Nature Portfolio policies, see our Editorial Policies and the Editorial Policy Checklist.

Statistics

For all statistical analyses, confirm that the following items are present in the figure legend, table legend, main text, or Methods section.

n/a	Confirmed	
☐	☒	The exact sample size (*n*) for each experimental group/condition, given as a discrete number and unit of measurement
☐	☒	A statement on whether measurements were taken from distinct samples or whether the same sample was measured repeatedly
☐	☒	The statistical test(s) used AND whether they are one- or two-sided<br>*Only common tests should be described solely by name; describe more complex techniques in the Methods section.*
☒	☐	A description of all covariates tested
☐	☒	A description of any assumptions or corrections, such as tests of normality and adjustment for multiple comparisons
☐	☒	A full description of the statistical parameters including central tendency (e.g. means) or other basic estimates (e.g. regression coefficient) AND variation (e.g. standard deviation) or associated estimates of uncertainty (e.g. confidence intervals)
☐	☒	For null hypothesis testing, the test statistic (e.g. *F*, *t*, *r*) with confidence intervals, effect sizes, degrees of freedom and *P* value noted<br>*Give P values as exact values whenever suitable.*
☒	☐	For Bayesian analysis, information on the choice of priors and Markov chain Monte Carlo settings
☒	☐	For hierarchical and complex designs, identification of the appropriate level for tests and full reporting of outcomes
☒	☐	Estimates of effect sizes (e.g. Cohen's *d*, Pearson's *r*), indicating how they were calculated

Our web collection on statistics for biologists contains articles on many of the points above.

Software and code

Policy information about availability of computer code

Data collection	Live cell imaging is performed using an Incucyte instrument. Flow cytometry analysis is performed using a Cytoflex S, LX or a BD Fortessa instrument. Western blotting images were captured using an Amersham Imager 680. For ELISA, absorbance at a wavelength of 450 nm and 570 nm was recorded using a CLARIOstar plus instrument. Label-free proteome quantification was performed using a Q-Extractive Plus Mass Spectrometer.
Data analysis	Genome-wide CRISPR screens were analyzed using the MaGeCK software. Differential protein expression in proteomics was calculated with R version 4.0.5. Proteins were queried for over representation analysis against the Reactome (17367534) and Gene Ontology Biological Process (10802651; 33290552) collections from the Molecular Signature Database using clusterProfiler (34557778). Flow cytometry data was analyzed using the FlowJo V10 software. Figures were generated using Graphpad Prism version 9 or 10. Protein interactions were visualized using STRING and Cytoscape V.3.9.1. CRISPR screen results were analyzed using the MAGeCKsoftware.

For manuscripts utilizing custom algorithms or software that are central to the research but not yet described in published literature, software must be made available to editors and reviewers. We strongly encourage code deposition in a community repository (e.g. GitHub). See the Nature Portfolio guidelines for submitting code & software for further information.

Data

Policy information about availability of data

All manuscripts must include a data availability statement. This statement should provide the following information, where applicable:

- Accession codes, unique identifiers, or web links for publicly available datasets
- A description of any restrictions on data availability
- For clinical datasets or third party data, please ensure that the statement adheres to our policy

The mass spectrometry proteomics data generated in this study have been deposited in the ProteomeXchange Consortium via the PRIDE partner repository under the identifier PXD051061. The processed proteomics results are included as Supplementary Data 2. Raw data from the Nanostring analsysis is included as Supplementary Data 3. The processed gRNA and gene level data are included as Supplementary Data 1. The publicly available large scale cell line CRISPR KO screen data (2022Q4 release) used in this study are available in the Cancer Dependency Map portal (DepMap) [https://depmap.org/portal]. Publicly available breast cancer patient data (NCT01042379) used in this study are available in the NCBI GEO database under accession code GSE194040 [https://www.ncbi.nlm.nih.gov/geo/query/acc.cgi?acc=GSE194040]. The remaining data are available within the Article, Supplementary Information or Source Data file.

Research involving human participants, their data, or biological material

Policy information about studies with human participants or human data. See also policy information about sex, gender (identity/presentation), and sexual orientation and race, ethnicity and racism.

Reporting on sex and gender	No human subjects were involved in the study.
Reporting on race, ethnicity, or other socially relevant groupings	*Please specify the socially constructed or socially relevant categorization variable(s) used in your manuscript and explain why they were used. Please note that such variables should not be used as proxies for other socially constructed/relevant variables (for example, race or ethnicity should not be used as a proxy for socioeconomic status).*<br>*Provide clear definitions of the relevant terms used, how they were provided (by the participants/respondents, the researchers, or third parties), and the method(s) used to classify people into the different categories (e.g. self-report, census or administrative data, social media data, etc.)*<br>*Please provide details about how you controlled for confounding variables in your analyses.*
Population characteristics	*Describe the covariate-relevant population characteristics of the human research participants (e.g. age, genotypic information, past and current diagnosis and treatment categories). If you filled out the behavioural & social sciences study design questions and have nothing to add here, write "See above."*
Recruitment	*Describe how participants were recruited. Outline any potential self-selection bias or other biases that may be present and how these are likely to impact results.*
Ethics oversight	*Identify the organization(s) that approved the study protocol.*

Note that full information on the approval of the study protocol must also be provided in the manuscript.

Field-specific reporting

Please select the one below that is the best fit for your research. If you are not sure, read the appropriate sections before making your selection.

☒ Life sciences ☐ Behavioural & social sciences ☐ Ecological, evolutionary & environmental sciences

For a reference copy of the document with all sections, see nature.com/documents/nr-reporting-summary-flat.pdf

Life sciences study design

All studies must disclose on these points even when the disclosure is negative.

Sample size	Sample size is determined based on pilot studies for each model and previous experimental evidence. No statistical method is used to determine sample size.
Data exclusions	For in vivo studies, mice developing ulcerations in subcutaneous tumors or other abnormality are excluded due to predefined humane endpoints. For functional assays using primary immune cells, donor cells show no response to stimulation or high background activation without stimulation are excluded.
Replication	For in vitro studies, at least 3 biological replicates/donors are included. Replications with inactive lymphocytes or high background activation were considered unsuccessful. For in vivo studies, at least two biological replicates are performed with 6-10 animals per experimental group. The exact percentages of mice with small or no tumor after anti-PD1 treatment differ among experiments but the strong response in NEDD8 KO tumors to anti-PD1 therapy remains consistent in all replications.

Randomization	For in vivo studies, mice are randomly allocated to receive wild type or NEDD8 KO cells and randomly allocated to control or treatment groups. For CRISPR screens or in vitro studies using primary immune cells from anonymous blood donors, random blood donors were used. For all in vitro assays, every biological repeat utilized cells from the same passage or blood donor. Compounds or proteins were then according to the microplates using a pre-designed platemap.
Blinding	For most in vivo studies, animal facility staff are asked to measure tumor volumes. They are blinded from the studies. For analysis of cells derived from tumor tissues, investigators were blinded during tissue collection. Blinding for other experimental procedures were not possible because group information was necessary for assay setup.

Reporting for specific materials, systems and methods

We require information from authors about some types of materials, experimental systems and methods used in many studies. Here, indicate whether each material, system or method listed is relevant to your study. If you are not sure if a list item applies to your research, read the appropriate section before selecting a response.

Materials & experimental systems

n/a	Involved in the study
☐	☒ Antibodies
☐	☒ Eukaryotic cell lines
☒	☐ Palaeontology and archaeology
☐	☒ Animals and other organisms
☒	☐ Clinical data
☒	☐ Dual use research of concern
☒	☐ Plants

Methods

n/a	Involved in the study
☒	☐ ChIP-seq
☐	☒ Flow cytometry
☒	☐ MRI-based neuroimaging

Antibodies

Antibodies used	Information regarding antibodies, e.g. clone number, catalog number, application, etc, is shown in Supplemental Table 1.
Validation	The primary antibodies detecting human and murine NEDD8 (Abcam), NEDP1 (Thermo Fisher) and NAE1 (Proteintech) were validated by CRISPR KO cells as shown in the figures. Antibody for CDT1 was validated using a compound treatment that is known to enhance its expression. Fluororescence-conjugated antibodies are selected from manufacturers that provide representative data and citations. For antibodies detecting well-studied lineage markers on immune cells, e.g. CD45, CD3, CD4, CD8 and CD56, detection of both positive and negative cell population in the same sample was required (supplementary figure 5b and 8a). Isotype control antibodies with the same fluorochrome conjugation were used in the paper to confirm the true positivity. For in vivo studies, the widely used anti-mPD1 clone, RMP1-14, was selected. The corresponding isotype control was used in the same study to ensure data validity. Approved therapeutic antibodies, i.e. nivolumab and durvalumab, were directly purchased from the pharmacy. No validation is required.

Eukaryotic cell lines

Policy information about cell lines and Sex and Gender in Research

Cell line source(s)	Human breast cancer cell line, MDA-MB-231 (92020424, Sigma Aldrich), and HEK293T cells (CRL-3216, American Type Culture Collection, ATCC) were purchased., were purchased from American Type Culture Collection (ATCC). HCC1937 and BT549 cell lines were a gift from Dr. Óscar Fernández-Capetillo (Karolinska Institutet, Sweden). Mouse breast cancer cell line EO771 was kindly provided by Dr. Maria Ulvmar (Uppsala University, Sweden).
Authentication	STR DNA fingerprinting was performed for all human cell lines by Eurofins.
Mycoplasma contamination	Mycoplasoma contamination is routinely tested by the MycoAlert kit and all cell lines were free from mycoplasma before used in the experiments.
Commonly misidentified lines (See ICLAC register)	Not identified.

Animals and other research organisms

Policy information about studies involving animals; ARRIVE guidelines recommended for reporting animal research, and Sex and Gender in Research

Laboratory animals	Six to ten weeks old female C57BL/6NTac or C57BL/6J mice were purchased from Taconic. All mice were housed in a barrier facility at the Rudbeck Laboratory (Uppsala University) with a humidity between 45 and 65% and an average temperature of 23 degrees. The dark/light cycle was fixed to 12 hours.
Wild animals	No wild animals were used.

Reporting on sex	Only female mice were used.
Field-collected samples	None of the samples were collected from field.
Ethics oversight	All animals were housed at the animal facility at the Department of Immunology, Genetics and Pathology in the Rudbeck laboratory at Uppsala University, and all studies were approved by the Swedish Board of Agriculture at Jönköping, Sweden (Dnr: 5.8.18-06394/2020).

Note that full information on the approval of the study protocol must also be provided in the manuscript.

Plants

Seed stocks	Not relevant.
Novel plant genotypes	*Describe the methods by which all novel plant genotypes were produced. This includes those generated by transgenic approaches, gene editing, chemical/radiation-based mutagenesis and hybridization. For transgenic lines, describe the transformation method, the number of independent lines analyzed and the generation upon which experiments were performed. For gene-edited lines, describe the editor used, the endogenous sequence targeted for editing, the targeting guide RNA sequence (if applicable) and how the editor was applied.*
Authentication	*Describe any authentication procedures for each seed stock used or novel genotype generated. Describe any experiments used to assess the effect of a mutation and, where applicable, how potential secondary effects (e.g. second site T-DNA insertions, mosiacism, off-target gene editing) were examined.*

Flow Cytometry

Plots

Confirm that:

- [x] The axis labels state the marker and fluorochrome used (e.g. CD4-FITC).
- [x] The axis scales are clearly visible. Include numbers along axes only for bottom left plot of group (a 'group' is an analysis of identical markers).
- [x] All plots are contour plots with outliers or pseudocolor plots.
- [x] A numerical value for number of cells or percentage (with statistics) is provided.

Methodology

Sample preparation	For in vitro assays, CTV-treated lymphocytes were harvested from TICS assay and transferred to a 96-well V bottom plate. The cells were centrifuged at 700×g for 4 min, followed by washing them twice with PBS. After that, cell pellets were resuspended in 20 µl PBS containing aqua fixable live/dead marker (Thermo Fisher Scientific) and then incubated at room temperature for 15 min. The cells were then washed twice with PBS and resuspended in 20 µl master mix containing detection antibodies for surface markers. After 20 min incubation at 4°C, the cells were washed and resuspended in 150 µl PBS for analysis. To determine the expression of immune related surface markers on NEDD8 KO and control cells, a multi-color flow cytometer was used. In brief, triple-negative control or NEDD8 KO breast cancer cells (5x105) were cultured in 6 well flat bottom plate in culture medium and incubated overnight to allow cells to attach. Following treatment with +/- rhIFNγ (50 ng/ml) for 24 hrs, cells were harvested, and centrifuged at 350×g for 4 min. Then, the cells were resuspended in 900 µl PBS and distributed in a 96 well V bottom plate in tritetraplicates (200 µl/well). Subsequently, the plate was centrifuged at 700×g for 4 min and resuspended in 20 µl PBS containing blue-fluorescent reactive dye (Thermo Fisher Scientific), detection antibodies for surface proteins (1:100) or the matching isotype control IgG for 25 min at 4°C. After being washed with PBS, the cells were resuspended in 150 µl PBS and transferred into FACS tubes for analysis.<br>For in vivo studies, single cells from tumor tissues were generated using a Tumor Dissociation Kit (Miltenyi Biotech) using the GentleMacs instrument according to the manufacturer's instructions. Subsequently, cells were loaded in a 96 well V bottom plate, and stained with 20 µl PBS containing an Auqa fixable live/dead marker (1:200) and a Fc receptor blocking antibody (1:100, Thermo Fisher Scientific). Cells were then washed with PBS and stained with 20 µl PBS containing antibodies for surface proteins (1:100) for 30 minutes at 4 degrees. To detect intracellular proteins including FoxP3 and TNFa, cells were fixed and permeabilized using a FoxP3/transcription factor staining buffer set (eBioscience) and incubated with fluorochrome-conjugated antibodies (1:50) for 45 minutes at 4 degrees. The rest of the cells were frozen and stored in -150° C until use.
Instrument	CytoFlex S or LX or BD Fortessa
Software	FlowJo V10
Cell population abundance	For sorting of B2M KO MDA-MB-231 cells, at least 5000 cells were obtained. Sorted cells were cultured and expanded before further experiments. For flow cytometric analysis, a minimum of 5000 cells in the target cell population were required.

Gating strategy	Gating strategy was included in supplementary figure 5b and 8a.

☒ Tick this box to confirm that a figure exemplifying the gating strategy is provided in the Supplementary Information.
