# Supplementary material for: Loss of NEDD8 in cancer cells causes vulnerability to immune checkpoint blockade in triple-negative breast cancer
Source: Nat Commun. 2024 Apr 27;15:3581. doi: 10.1038/s41467-024-47987-x (PMC11055868; doi:10.1038/s41467-024-47987-x)
Supplement: Supplementary file 8 — Source Data [file 41467_2024_47987_MOESM8_ESM.zip › Papakyriacou_Source Data files/Uncut membranes for WB_R3.pptx]

## Slide 1
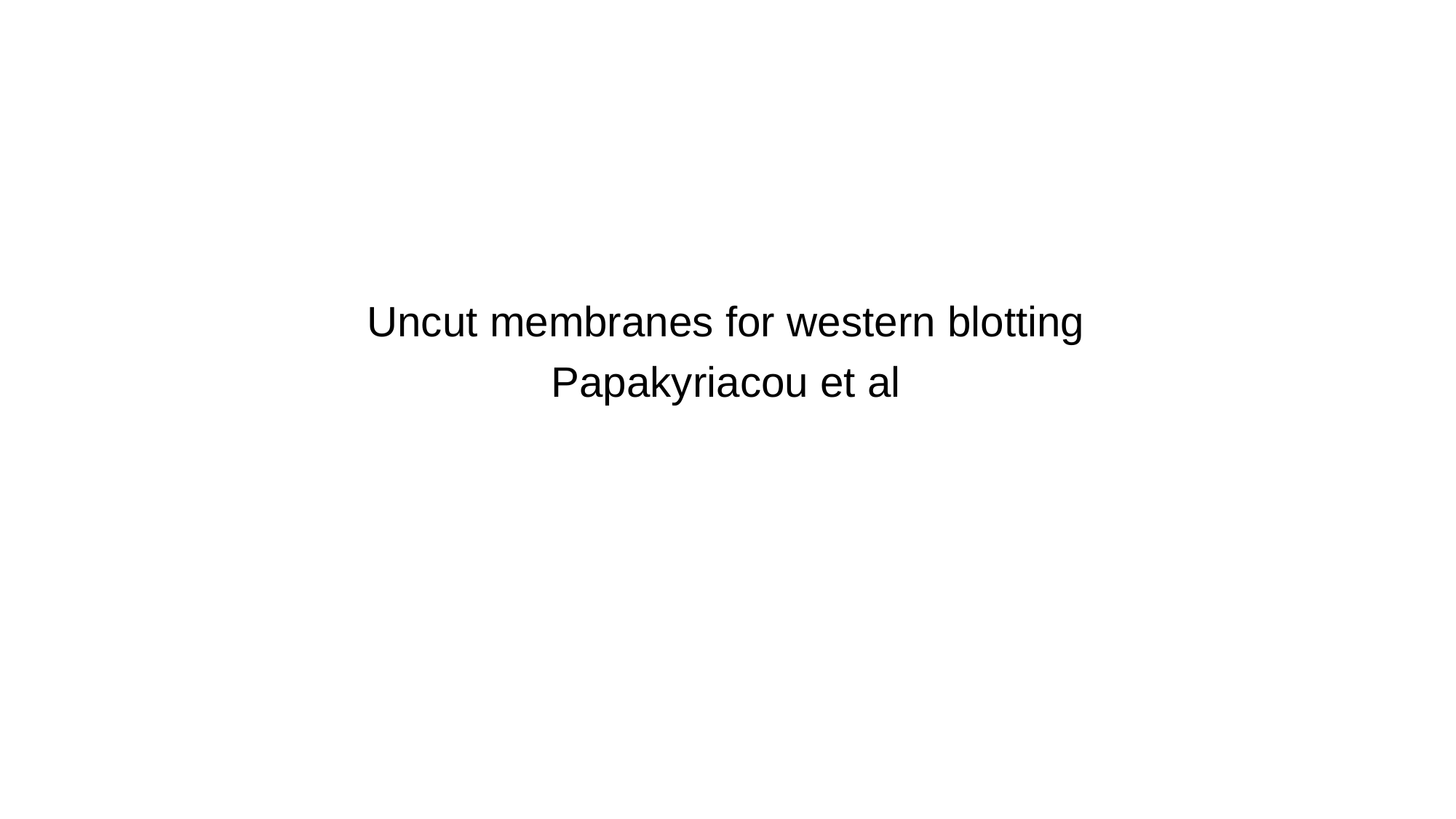

Uncut membranes for western blotting
Papakyriacou et al

## Slide 2
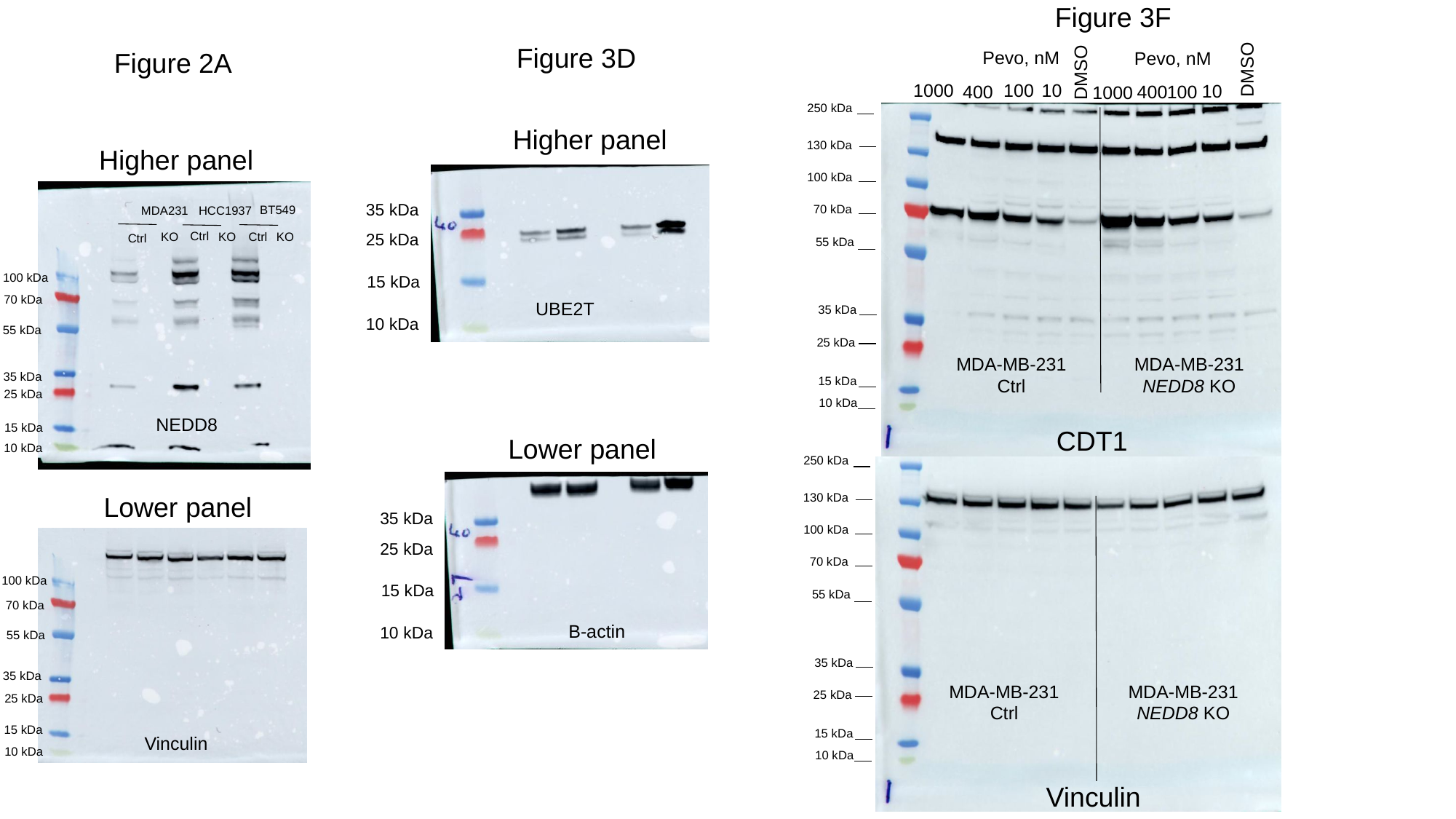

Figure 3F
Figure 3D
Figure 2A
Pevo, nM
Pevo, nM
DMSO
DMSO
100
10
1000
10
400
100
400
1000
250 kDa
Higher panel
130 kDa
Higher panel
100 kDa
35 kDa
25 kDa
15 kDa
UBE2T
10 kDa
B-actin
BT549
MDA231
HCC1937
Ctrl
KO
KO
KO
Ctrl
Ctrl
NEDD8
Vinculin
70 kDa
55 kDa
100 kDa
70 kDa
35 kDa
55 kDa
25 kDa
MDA-MB-231
Ctrl
MDA-MB-231
NEDD8 KO
35 kDa
15 kDa
25 kDa
10 kDa
15 kDa
CDT1
Lower panel
10 kDa
250 kDa
Lower panel
130 kDa
35 kDa
100 kDa
25 kDa
70 kDa
100 kDa
15 kDa
55 kDa
70 kDa
10 kDa
55 kDa
35 kDa
35 kDa
MDA-MB-231
Ctrl
MDA-MB-231
NEDD8 KO
25 kDa
25 kDa
15 kDa
15 kDa
10 kDa
10 kDa
Vinculin

## Slide 3
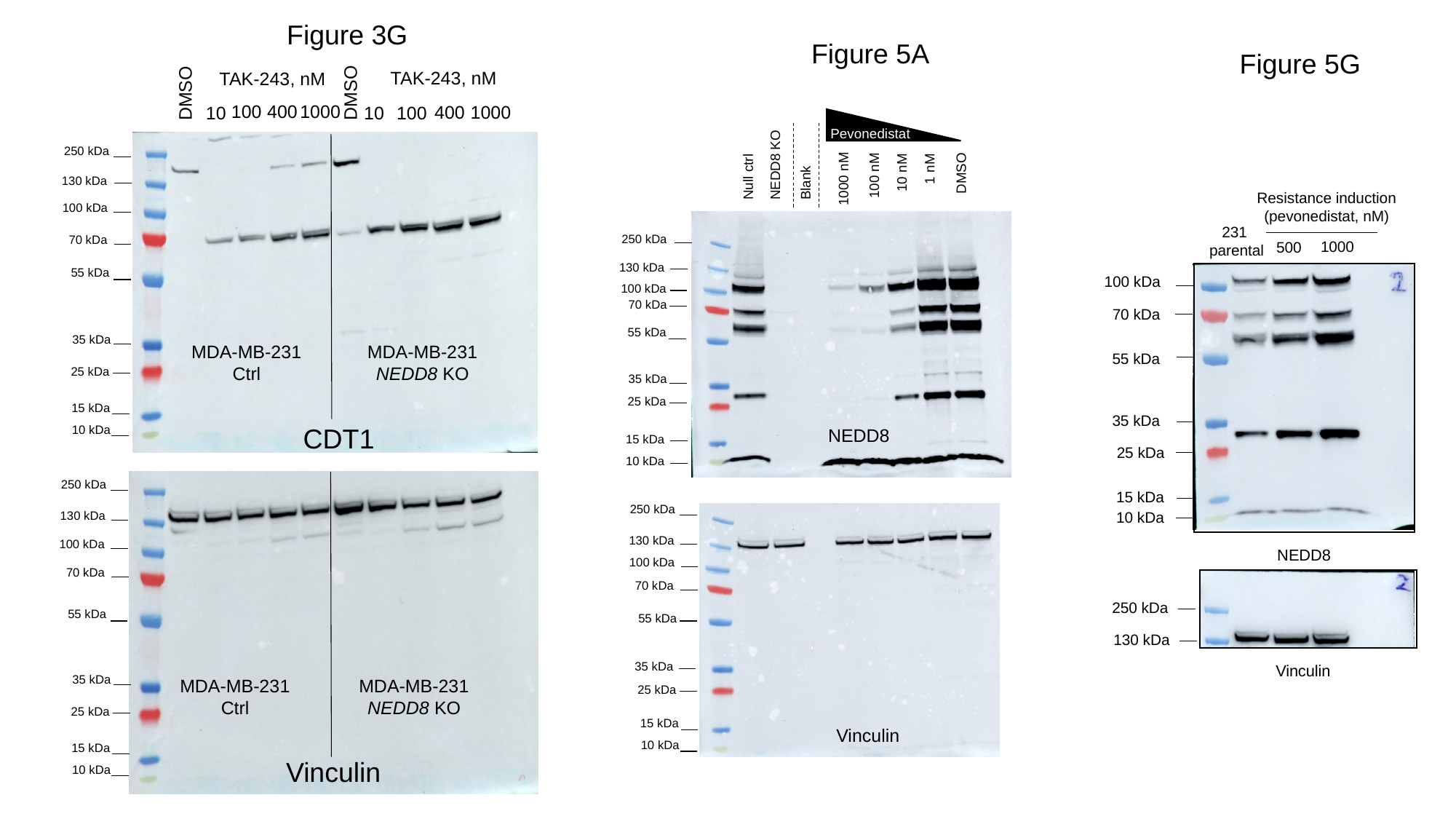

Figure 3G
Figure 5A
Figure 5G
TAK-243, nM
TAK-243, nM
DMSO
DMSO
1000
400
100
400
1000
10
100
10
Pevonedistat
1 nM
DMSO
130 kDa
100 kDa
70 kDa
55 kDa
35 kDa
25 kDa
NEDD8
NEDD8 KO
Blank
Null ctrl
1000 nM
10 nM
100 nM
15 kDa
10 kDa
Vinculin
250 kDa
130 kDa
Resistance induction
(pevonedistat, nM)
231
parental
1000
500
100 kDa
70 kDa
55 kDa
35 kDa
25 kDa
10 kDa
NEDD8
130 kDa
Vinculin
100 kDa
250 kDa
70 kDa
55 kDa
35 kDa
MDA-MB-231
Ctrl
MDA-MB-231
NEDD8 KO
25 kDa
15 kDa
CDT1
10 kDa
250 kDa
15 kDa
250 kDa
130 kDa
130 kDa
100 kDa
100 kDa
70 kDa
70 kDa
250 kDa
55 kDa
55 kDa
35 kDa
35 kDa
MDA-MB-231
Ctrl
MDA-MB-231
NEDD8 KO
25 kDa
25 kDa
15 kDa
10 kDa
15 kDa
Vinculin
10 kDa

## Slide 4
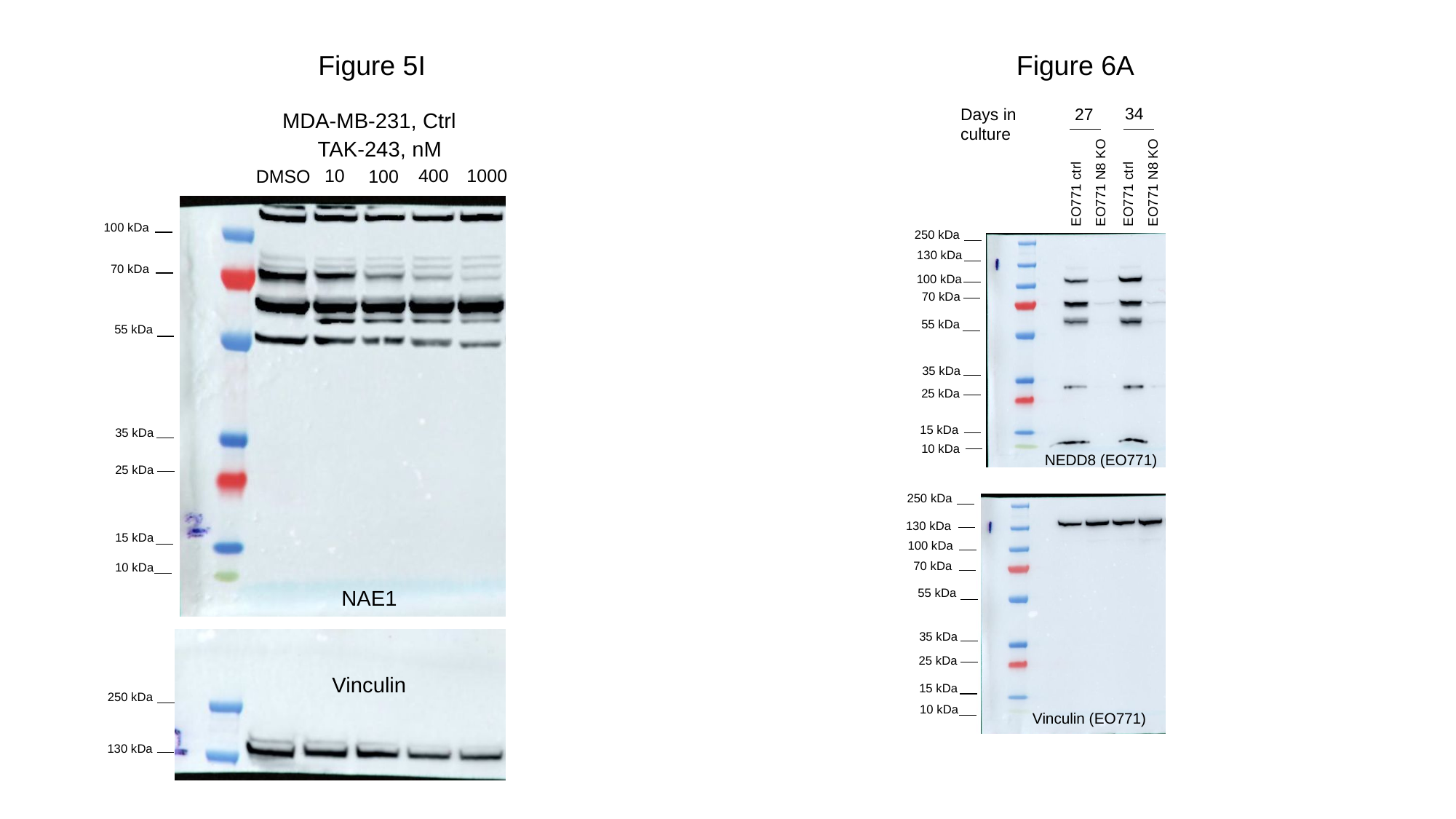

Figure 5I
Figure 6A
34
Days in culture
27
EO771 N8 KO
EO771 ctrl
130 kDa
100 kDa
70 kDa
55 kDa
35 kDa
25 kDa
NEDD8 (EO771)
15 kDa
10 kDa
Vinculin (EO771)
EO771 N8 KO
EO771 ctrl
MDA-MB-231, Ctrl
TAK-243, nM
10
1000
400
DMSO
100
100 kDa
250 kDa
70 kDa
55 kDa
35 kDa
25 kDa
250 kDa
130 kDa
15 kDa
100 kDa
70 kDa
10 kDa
NAE1
55 kDa
35 kDa
25 kDa
Vinculin
15 kDa
250 kDa
10 kDa
130 kDa

## Slide 5
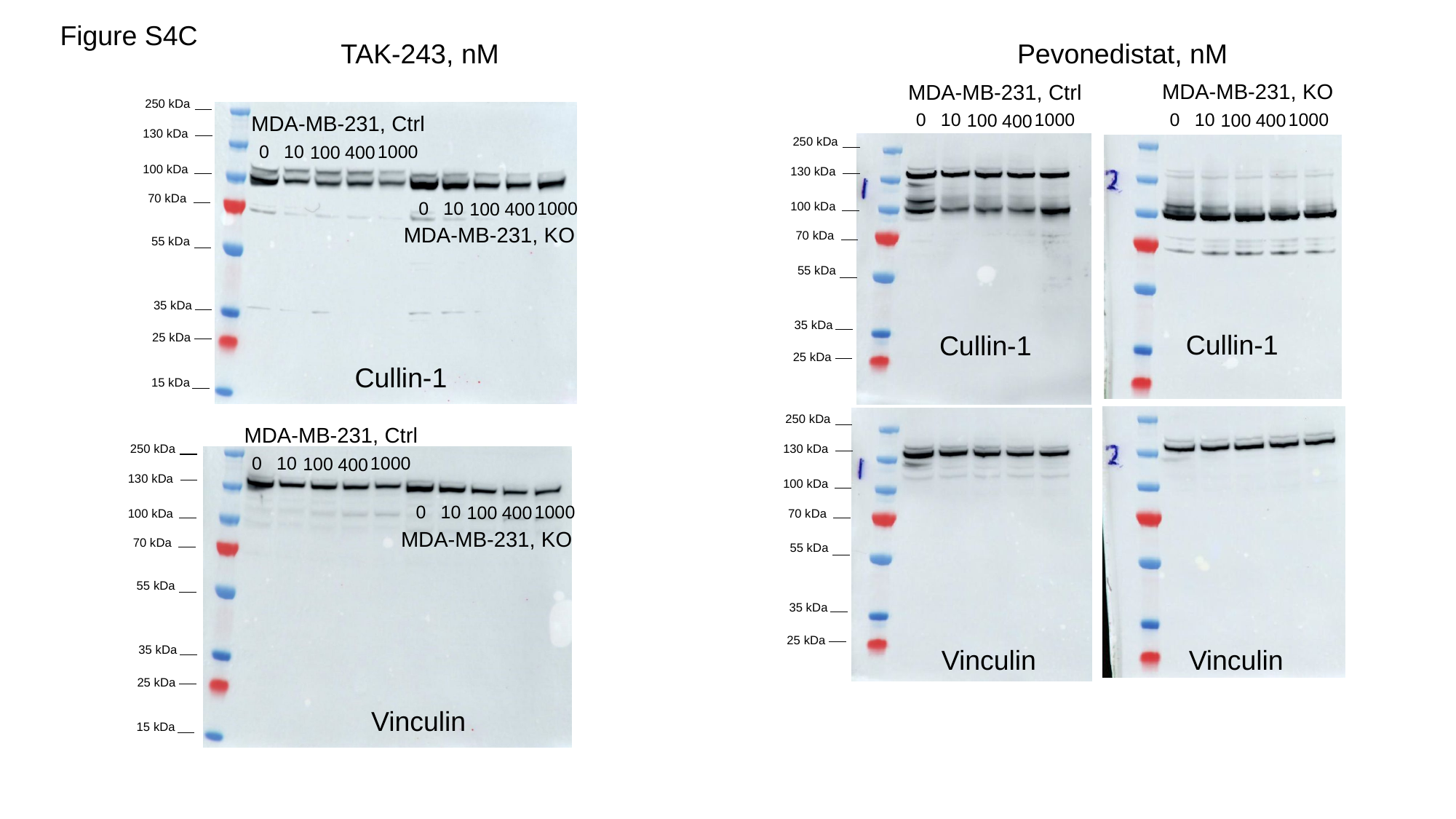

Figure S4C
TAK-243, nM
Pevonedistat, nM
MDA-MB-231, KO
MDA-MB-231, Ctrl
250 kDa
10
1000
0
10
1000
0
100
400
100
400
MDA-MB-231, Ctrl
130 kDa
250 kDa
10
1000
0
100
400
100 kDa
130 kDa
70 kDa
10
1000
0
100
400
100 kDa
MDA-MB-231, KO
70 kDa
55 kDa
55 kDa
35 kDa
35 kDa
Cullin-1
Cullin-1
25 kDa
25 kDa
Cullin-1
15 kDa
250 kDa
MDA-MB-231, Ctrl
250 kDa
130 kDa
10
1000
0
100
400
130 kDa
100 kDa
10
1000
0
100
400
70 kDa
100 kDa
MDA-MB-231, KO
70 kDa
55 kDa
55 kDa
35 kDa
25 kDa
35 kDa
Vinculin
Vinculin
25 kDa
Vinculin
15 kDa
